# Metastatic Sigmoid Colon Malignancy With a Synchronous Carcinoma Breast: Is Cure Possible?

**DOI:** 10.7759/cureus.21660

**Published:** 2022-01-27

**Authors:** Srikanth Gadiyaram, Murugappan Nachiappan, Ravikiran Thota

**Affiliations:** 1 Department of Surgical Gastroenterology and Minimally Invasive Surgery, Sahasra Hospitals, Bangalore, IND

**Keywords:** metastasis, breast cancer, sigmoid cancer, synchronous, multiple primary malignancies

## Abstract

Malignancies developing in two organs or more in the same patient are called multiple primary malignancies. They can be synchronous or metachronous based on the time of diagnosis of second cancer from the first. We encountered a synchronous stage IV sigmoid colon cancer (resectable liver metastasis) and breast cancer in a lady. The clinical dilemmas that arose with multiple primary malignancies and how they were tackled in our case have been discussed. A second malignancy should not deter the management or alter the clinical decision-making. Multidisciplinary teams are crucial to the management of these rare occurrences. We could successfully manage a synchronous breast and colon cancer with resectable liver metastasis at presentation.

## Introduction

Multiple primary malignancies are tumors in two or more organs in the same patient. They could be synchronous or metachronous [1]. The Surveillance, Epidemiology, and End Results (SEER) database analysis of 4835 women with both breast and colon cancers reported synchronous cancers in 3.8% of the patients [2]. Even though the role of surgery, chemotherapy and radiotherapy in the management of these individual malignancies is well defined, decision-making becomes difficult in the setting of synchronous malignancies. The treating team faces management dilemmas pertaining to chemotherapy and radiotherapy regimens in the neoadjuvant setting, such as the timing of surgery for the individual malignancies, whether a simultaneous resection could be undertaken, the use of adjuvant therapy, the follow-up strategies, etc. The entire gamut of problems could be further complicated by a nihilistic attitude in the setting of a synchronous malignancy, particularly in the elderly. We report the management of a lady with synchronous colon cancer (stage IV, resectable liver metastasis) and breast cancer (stage I). The relevant issues in the management are addressed with the help of the literature data available on the topic.

## Case presentation

**Figure 1:**
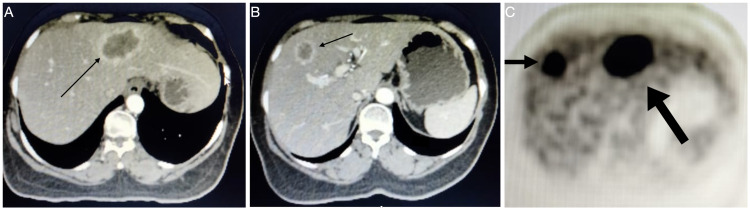
PET-CT images at initial presentation, (A) Metastatic lesion in the segment II/III of the liver (line arrow) which required a left lateral segmentectomy, (B) Metastatic lesion in the segment IVa/VIII of the liver (line arrow), which was ablated, and (C) PET image demonstrating FDG uptake in both the lesions in segment II/III (long block arrow) and segment IVa/VIII (short block arrow). PET: Positron emission tomography; FDG: Fluorodeoxyglucose.

**Figure 2:**
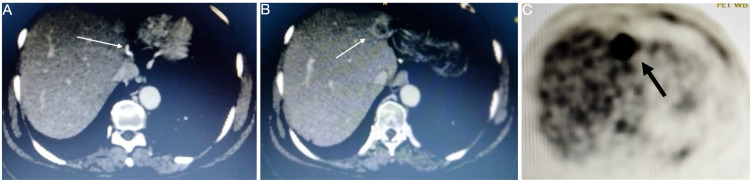
(A) Follow-up CT after left lateral segmentectomy showing surgical staples (line arrow) applied to the left hepatic vein, (B) Recurrence of the lesion in the segment IVa (line arrow), and (C) Corresponding PET image showing FDG uptake in the lesion (black arrow). PET: Positron emission tomography; FDG: Fluorodeoxyglucose.

A 57-year-old lady with known hypothyroidism presented to us with complaints of abdominal pain and constipation. There was no family history of malignancy. The abdominal (rectal) examination was normal. Sigmoidoscopy revealed an obstructing growth in the sigmoid colon, and ultrasound showed two liver metastases in the left lobe of the liver. Biopsy of the sigmoid growth suggested moderately differentiated adenocarcinoma. Staging positron emission tomography (PET) CT suggested metastatic sigmoid adenocarcinoma (cT3+M1a). There were a few mesocolic nodes of max diameter 11 mm without fluorodeoxyglucose (FDG) uptake, along with two lesions in the liver - larger one in segment II, III (58 mm * 35 mm, standard uptake value [SUVmax] 22.9) and another in segment IVA/VIII (27 mm * 25 mm, SUVmax 19.3) (Figure 1A-1C). PET CT also showed a 15 mm * 10 mm lesion with a SUVmax of 4.9 in the upper inner quadrant of the left breast. Fine needle aspiration cytology was negative for malignancy. The patient was a carcinoembryonic antigen (CEA) nonsecretor. After discussion in a multidisciplinary tumor board, the patient was planned for laparoscopic sigmoid colectomy in view of impending obstruction, followed by chemotherapy for the liver metastasis and further plan based on response to chemotherapy. The procedure was uneventful, and the hospital stay was five days (pT3N0M1a). The patient received four cycles of FOLFOX chemotherapy. Follow-up PET showed significant interval regression in the size of the lesions in segment II/III to 24 mm * 21 mm and segment IVA/VIII lesion to 10 mm * 16 mm with a resolution of FDG uptake. The patient was planned for laparoscopic left lateral segmentectomy along with radiofrequency ablation of the lesion in the segment IVA/VIII. The procedure was uneventful, with a hospital stay of four days. The patient completed six further cycles of chemotherapy and tolerated it well. Follow-up PET at nine months showed no recurrence of disease; however, the lesion in the left breast had similar findings to the previous scan. The patient was taken up for wide local excision of the breast lump, which suggested infiltrative ductal adenocarcinoma. Following this, the patient underwent a completion mastectomy with axillary clearance (pT1N0M0). The tumor was estrogen receptor (ER), progesterone receptor (PR) positive, Her-2/Neu negative, and Ki-67 index ≤15%. The patient was offered hormonal therapy with an aromatase inhibitor. At one and a half years follow up, there was a recurrence in segment IVA (22 mm * 19 mm, SUVmax 19.3) (Figure 2A-2C). The patient underwent radiofrequency ablation of the lesion, and later a laparoscopic completion left hepatectomy for a persistent lesion after three cycles of FOLFIRI. The patient is asymptomatic and doing well six years from the index diagnosis (three years from the last surgery). She has completed hormonal therapy for breast cancer.

## Discussion

Synchronous multiple primary cancers are cancers of two different origins that are identified simultaneously or within six months of diagnosis of one tumor [1]. There can be a lead time bias in this, as in our case, where the breast cancer was identified because of the staging workup done for sigmoid malignancy. The patient's prognosis depends on the tumor stages of the cancers independently. Thus, with adequate treatment, the prognosis should not be worse than individual tumors [3]. In a SEER database analysis of 4835 women with both breast and colon cancers, the incidence of synchronous cancer was 3.8% [2]. It was found that the calculated death rates were higher for colorectal cancer than breast cancer. Colorectal cancer was found to cause death nearly three times more than breast cancer. Colorectal cancer-specific mortality increased with time, whereas breast cancer-specific mortality decreased with time [2]. These findings should be kept in mind while making treatment decisions in these patients. The pertinent questions to be answered when dealing with a synchronous cancer are as follows. First, if a cure is possible when these cancers were seen in isolation, then towards which one should the treatment strategy in terms of neo-adjuvant therapy and surgery be directed? Synchronous malignancies pose a decision-making problem. Acceptable and reasonable clinical decision-making would suggest that the therapy is guided by the more advanced or more aggressive tumor. Some groups opine that a less morbid surgery should be undertaken initially to complete the next surgery in a shorter time frame [4]. Simultaneous resections for both malignancies have been reported by a few [5,6].

Second, having decided which one needs to be targeted, what should be the treatment plan, and how is it different from the one should this cancer have occurred in isolation? Regarding the stage IV colon cancer with liver metastasis at presentation, in the multidisciplinary tumor board, it was decided that the colon cancer will be addressed first. With synchronous liver metastasis, neoadjuvant chemotherapy would have been considered as the initial modality. However, given the non-negotiable tumor on sigmoidoscopy, the patient was taken up for sigmoid resection first, followed by chemotherapy for liver metastasis and liver resection later on. This treatment plan would have remained essentially the same irrespective of the diagnosis of synchronous breast cancer.

Third, would the adjuvant chemotherapy in such a patient be different or extended than a patient who had cancer in isolation? Few of the reports that have discussed the adjuvant therapy have mentioned no change in the protocol, and the systemic therapy was directed to the malignancy with an advanced stage [4,7]. Similarly, our patient had breast cancer with no poor prognostic factor hence only hormonal therapy was considered. In contrast, she had finished her protocol of doublet chemotherapy for stage IV colon cancer. Finding blended chemotherapy, which can target both the diseases in cases that require adjuvant systemic therapy without significant toxicity, could be challenging. Thus, this issue must be discussed in a multidisciplinary tumor board, and treatment must be individualized.

Last, with a curative treatment achieved for both, what should be the treatment of recurrent liver metastasis in such a patient? With a reported five-year survival of 30-40% in patients undergoing re-resection, a patient with good performance status with adequate hepatic function, it should be considered if the recurrence is resectable [8]. In an unresectable liver metastasis or distant disease, nonsurgical regional therapies and palliation have to be considered.

## Conclusions

The management of patients with synchronous cancers needs to be individualized. If the individual cancers are curable based on the staging, then the overall management intent must be curative. This needs to be guided by a multidisciplinary team. A second malignancy should not be a deterrent for treatment. Multidisciplinary teams play a crucial role and help in decision-making. For example, we could achieve a cure in a case of synchronous breast cancer and metastatic colon cancer with resectable liver metastasis by using multi-modality treatment.
